# TrkA inhibitor promotes motor functional regeneration of recurrent laryngeal nerve by suppression of sensory nerve regeneration

**DOI:** 10.1038/s41598-020-72288-w

**Published:** 2020-10-09

**Authors:** Hiroshi Suzuki, Koji Araki, Toshiyasu Matsui, Yuya Tanaka, Kosuke Uno, Masayuki Tomifuji, Taku Yamashita, Yasushi Satoh, Yasushi Kobayashi, Akihiro Shiotani

**Affiliations:** 1 Department of Otolaryngology-Head and Neck Surgery, National Defense Medical College, 3-2 Namiki, Tokorozawa, Saitama 359-8513 Japan; 2 Department of Otolaryngology, Self-Defense Forces Central Hospital, Tokyo, Japan; 3 Department of Anatomy and Neurobiology, National Defense Medical College, Tokorozawa, Japan; 4 Laboratory of Veterinary Anatomy, Faculty of Veterinary Medicine, Okayama University of Science, Imabari, Japan; 5 Department of Otolaryngology-Head and Neck Surgery, Kitasato University School of Medicine, Sagamihara, Japan; 6 Department of Biochemistry, National Defense Medical College, Tokorozawa, Japan

**Keywords:** Motor control, Neurotrophic factors, Peripheral nervous system, Regeneration and repair in the nervous system

## Abstract

Recurrent laryngeal nerve (RLN) injury, in which hoarseness and dysphagia arise as a result of impaired vocal fold movement, is a serious complication. Misdirected regeneration is an issue for functional regeneration. In this study, we demonstrated the effect of TrkA inhibitors, which blocks the NGF-TrkA pathway that acts on the sensory/automatic nerves thus preventing misdirected regeneration among motor and sensory nerves, and thereby promoting the regeneration of motor neurons to achieve functional recovery. RLN axotomy rat models were used in this study, in which cut ends of the nerve were bridged with polyglycolic acid-collagen tube with and without TrkA inhibitor (TrkAi) infiltration. Our study revealed significant improvement in motor nerve fiber regeneration and function, in assessment of vocal fold movement, myelinated nerve regeneration, compound muscle action potential, and prevention of laryngeal muscle atrophy. Retrograde labeling demonstrated fewer labeled neurons in the vagus ganglion, which confirmed reduced misdirected regeneration among motor and sensory fibers, and a change in distribution of the labeled neurons in the nucleus ambiguus. Our study demonstrated that TrkAi have a strong potential for clinical application in the treatment of RLN injury.

## Introduction

Recurrent laryngeal nerve (RLN) injury, in which voice hoarseness and dysphagia arise as a result of impaired vocal fold movement, is a serious illness that can threaten patients’ quality of life as a result of aphonia and aspiration pneumonitis. RLN paralysis can be idiopathic or caused by factors such as surgery or a malignant tumour^1^. Surgical treatment for this condition focuses on static improvement of glottis closure via procedures such as injection laryngoplasty^2^, thyroplasty, or arytenoid adduction^3^. Some reports have described the use of intraoperative anastomoses with the ansa cervicalis, ansa hypoglossi, and phrenic nerve^4–7^. However, although laryngeal muscle atrophy could be prevented, vocal fold motor function could not be recovered.

The causes of vocal fold immobility following RLN injury include motor neuron death in the nucleus ambiguus, degeneration and poor regeneration of nerve fibres and motor endplates, laryngeal muscle atrophy, and misdirected regeneration^8–11^. Basic studies have shown that the former three can be overcome by using techniques such as gene therapy^12–17^. However, misdirected regeneration is not overcome and, this appears to be the most important issue.

We identified two major problems associated with misdirected regeneration^10^. The first is the fact that the RLN controls both the glottic adductors and abductors.

Misdirected regeneration among the motor fibers that control these muscles precludes correct closure and opening of the glottis in response to accurate commands, resulting in vocal fold immobility and paradoxical movement. The second problem is that the RLN is composed of motor, sensory, and automatic^18^. When sensory/automatic fibers connect to laryngeal muscles, the excitation of motor neurons does not transmit to the muscles and it prevents appropriate muscle activities. Thus, misdirected nerves can be avoided by eliminating misdirected regeneration of (1) abductor and adductor motor fibers, and (2) motor fibers and sensory/automatic fibers.

During post-axotomy nerve regeneration, misdirected regeneration occurs in motor fibers and sensory fibers in addition to that in motor fibers, which is thought to be one factor impeding vocal fold function recovery^19–22^. Therefore, we hypothesized that inhibition of sensory fiber regeneration could promote regeneration of motor fiberfibers, and investigated a method to eliminate the misdirected regeneration described in (2) motor fibers and sensory/automatic fibers.

The neurotrophic family is composed of four neurotrophic factors. Nerve growth factor (NGF), which is one of the neurotrophic factor family, binds with high-affinity to TrkA, and with low affinity to p75 neurotrophic receptors^22,23^. Pathways located downstream from TrkA are thought to affect sensory fiber and automatic fiber regeneration^24^ (Supplementary Fig. 1). Therefore, we thought that selective inhibition of the TrkA pathway could selectively impede the misdirected regeneration of motor and sensory/automatic fibers, and we focused on TrkA inhibitors.

**Figure 1:**
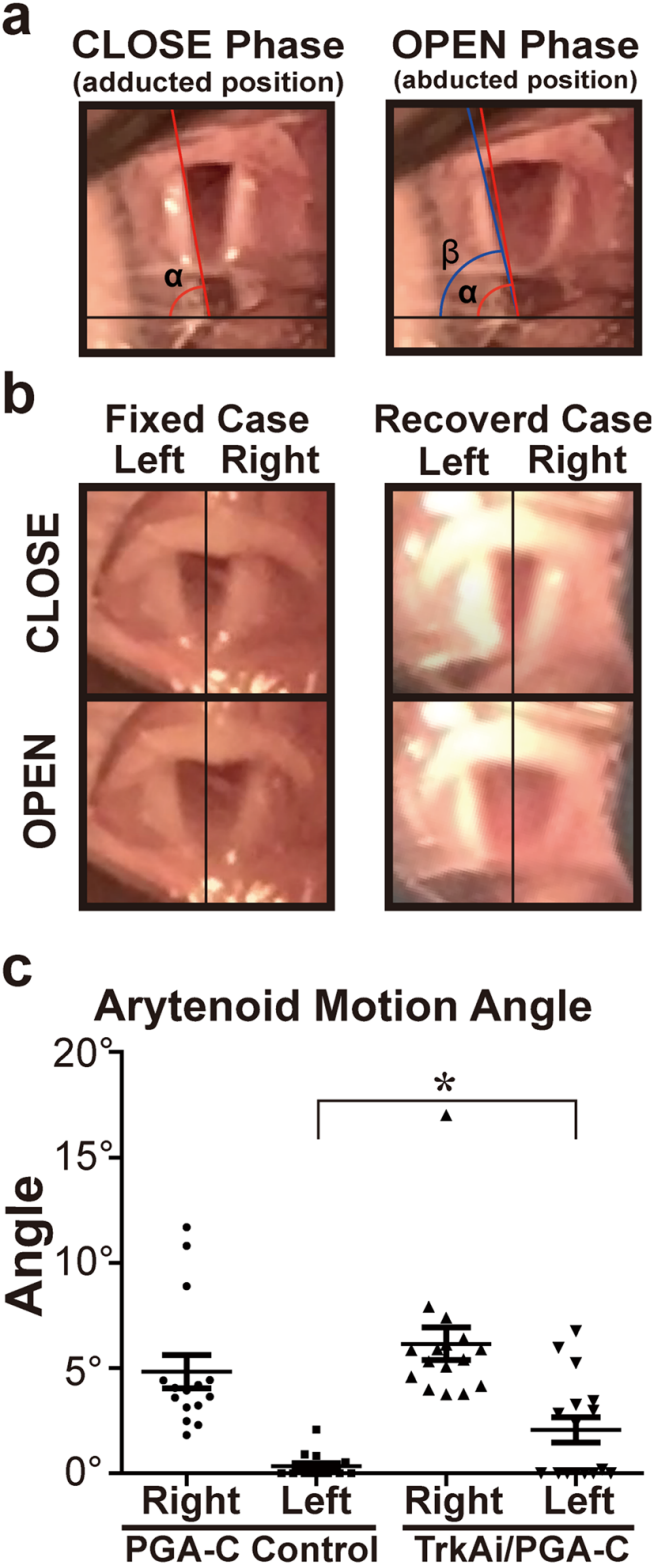
Assessment of left arytenoid mobility at 14 weeks after surgery. (**a**) Images of arytenoid angles in the CLOSE phase (maximal adducted position) and the OPEN phase (maximal abducted position). The baseline was defined as a horizontal line through the intersection of the left and right arytenoid lines in the CLOSE phase. The difference between the maximal adducted (α) and abducted (β) angles was defined as the arytenoid motion angle. Blue line: maximal abducted arytenoid line, black line: baseline. (**b**) Representative findings of vocal fold mobility. The fixed case (left columns, arytenoid motion angle < 1°) (Video 1) and the recovered case (right columns, arytenoid motion angle > 5°) (Video 2) of the left arytenoid cartilage are shown. The upper columns are in the CLOSE phase and the lower columns are in the OPEN phase. (**c**) Degree of arytenoid motion angle in the PGA-C Control and the TrkAi/PGA-C. In left arytenoid motion angles, there was a significant difference between the two groups. *p < 0.05.

In this study, using a left RLN axotomy model that we had previously reported^25^, we investigated whether treatment with TrkA inhibitors improved vocal fold function. As a basis for polyglycolic acid (PGA)-collagen tube treatment we administered TrkA inhibitors and investigated improvements in vocal fold mobility, morphologic and electrophysiological properties of nerves, reduction in vocal fold atrophy, and suppression of misdirected regeneration in the central nervous system via evaluation with a retrograde tracer.

## Results

### TrkA inhibitor contributed to improvement of vocal fold movement

At 14 weeks post-surgery, vocal fold movement was assessed by determining the left arytenoid motion angles in the adducted position and abducted position^26^ (Fig. 1a). The representative findings for vocal fold mobility are shown in Fig. 1b, Video 1 and 2. The average right arytenoid motion angles in the PGA-Collagen tube bridging control group (PGA-C Control) (n = 15) and TrkA inhibitor-infiltrated PGA-C tube bridging treatment group (TrkAi/PGA-C) (n = 16) were 4.84° ± 0.79° and 6.15° ± 0.78°, respectively. The average left arytenoid motion angles in the PGA-C Control and TrkAi/PGA-C were 0.34° ± 0.15° and 2.07° ± 0.60°, respectively. There was a significant difference between the two groups in the left angles (Fig. 1c). An arytenoid motion angle more than 1° was considered to indicate positive mobility. The recovery rate of vocal fold movement was 7% (1/15) in PGA-C Control and 50% (8/16) in TrkAi/PGA-C (odds ratio: 14.00, 95% Cl: 1.470–133.3; *p* < 0.05). Three rats in TrkAi/PGA-C showed left arytenoid motion angle recovery of more than 5°, with the recovered left arytenoid showing almost the same mobility as the right arytenoid. One rat showed paradoxical arytenoid motion. TrkA inhibitor contributed to the recovery of vocal fold movement after RLN transection.

### Neurofunctional recovery was improved by TrkA inhibitor

**Figure 2:**
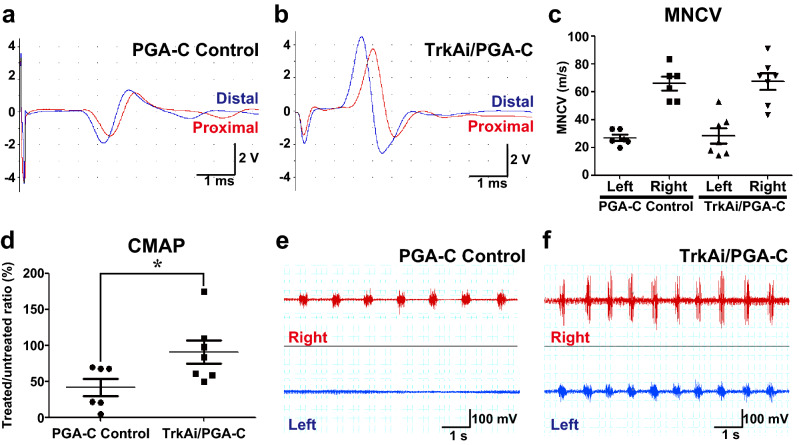
Electromyography (EMG) of the posterior cricoarytenoid (PCA) muscles 15 weeks after the procedure. (**a**,**b**) Representative EMG of the PCA muscles under stimulation of left RLNs of PGA-C Control (**a**) and TrkAi/PGA-C (**b**). Red waves were stimulated at the point 5 mm proximal from the surgical site. Blue waves were stimulated at the point 5 mm distal from the surgical site. (**c**) The motor nerve conduction velocities (MNCVs) of the right RLN (untreated-control side) and left RLN (treated side) were assessed in PGA-C Control and TrkAi/PGA-C. The normal control and treated sides were compared in both groups, but there was no significant difference. ns = *p* > 0.05 (Student’s *t* test). (**d**) The compound muscle action potentials (CMAPs) in PGA-C Control and TrkAi/PGA-C were compared by determining the treated/untreated CMAP ratio. There was a significant difference between the two groups. *p < 0.05. (**e**,**f**) The resting potentials of both right and left PCA muscles in PGA-C (**e**) and TrkAi/PGA-C (**f**). Upper red waves were the resting potential of the right PCA muscle and lower blue waves were the left PCA muscle. (**e**) In PGA-C Control, the resting potential waves of left PCA were not synchronized with right waves. (**f**) In PGA-C/TrkAi, only two cases showed resting potential wave synchronized with the right-side wave.

To validate neurofunctional recovery, we performed an EMG analysis of the PCA muscles at 15 weeks after the procedure, as reported previously^17,25^. The average MNCVs of the left RLNs recovered to about half of those on the right side in both groups. There were no differences between PGA-C Control and TrkAi/PGA-C (Fig. 2a–c). The average treated/untreated MNCV ratios did not differ as well. The CMAPs of the left RLNs in TrkAi/PGA-C were higher than those in the PGA-C Control (Fig. 2a, b). The average treated/untreated CMAP ratios were also significantly different between the two groups (Fig. 2d).

In respiratory synchronized electromyography of the right and left PCA muscles, synchronization of the left resting potential waves with right waves was not observed in PGA-C Control (Fig. 2e) but was observed in two rats in TrkAi/PGA-C (Fig. 2f). Although the MNCV did not show any improvement, it was possible that the improvement in CMAP contributed to the recovery of vocal fold movement.

### The TrkA inhibitor facilitated RLN myelinated fiber regeneration

**Figure 3:**
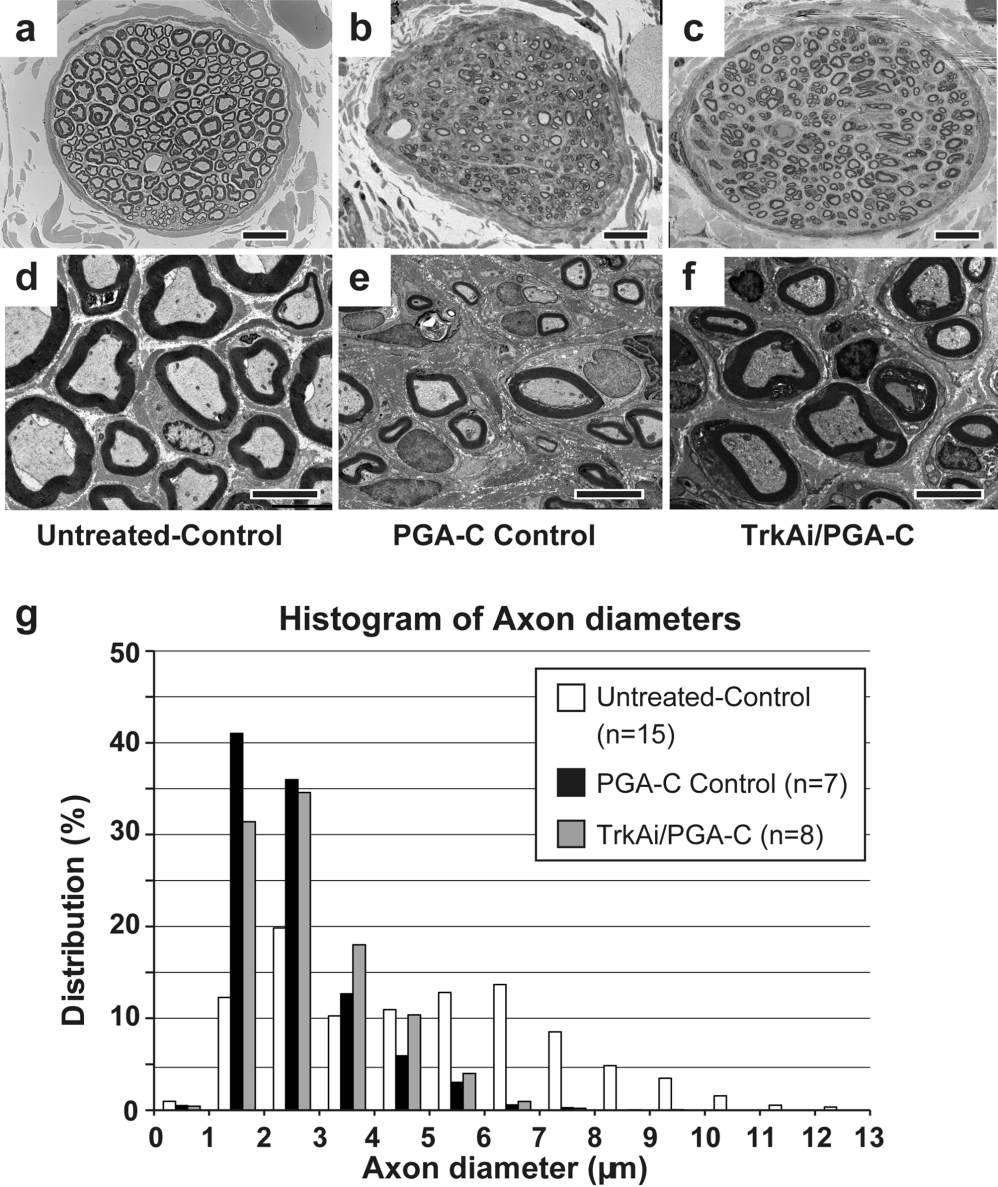
Electron microscopic findings of regenerated recurrent laryngeal nerves (RLNs). (**a**–**f**) Transverse sections of RLN distal to the treated site. (**a**,**d**) Normal control RLN (right RLN). (**b**,**e**) Left RLN in the PGA-C Control. (**c**,**f**) Left RLN in the TrkAi/PGA-C. A larger number of thicker myelinated fibers were observed in TrkAi/PGA-C (**c**,**f**) than in PGA-C Control (**a**,**e**). The scale bars are 20 µm for Panels **a**–**c** and 2 nm for **d**–**f**. (**g**) In the histogram of the diameters of myelinated axons, the distribution of axon diameters was compared in the untreated-control side (right RLN, white bar) and treated side (left RLN) of PGA-C Control (black bar) and TrkAi/PGA-C (gray bar). The distribution of axon diameters in TrkAi/PGA-C had shifted to the right more than in the PGA-C Control.

At 15 weeks post-surgery, nerve fiber connection at the surgical site was observed macroscopically in both groups. For morphological analysis of regenerated nerves, harvested RLNs were observed using electron microscopy^25,27^. In electron microscopic observation, TrkAi/PGA-C showed more and thicker myelinated fibers than PGA-C Control (Fig. 3a–c). In the high-magnification images obtained with electron microscopy, both groups showed regenerating and mature axons with myelin sheath formation and Schwann cells around the axons, but the number of regenerated myelinated axons was higher in TrkAi/PGA-C (Fig. 3d–f).

The morphological analysis (Table 1), showed that the number of myelinated fibersfibres in the left RLNs in TrkAi/PGA-C was higher than that in the PGA-C Control. The average diameters of the left myelinated axons in TrkAi/PGA-C were thicker than those in the PGA-C Control. While the histogram analysis of myelinated axons showed that the thicker axons in TrkAi/PGA-C were greater in number than those in the PGA-C Control (Fig. 3g). Thus, better regeneration of myelinated fibers was observed in TrkAi/PGA-C than in PGA-C control.

**Table 1:** Morphological data summary for the recurrent laryngeal nerves (RLNs).

	PGA-C Control (n = 7)	TrkAi/PGA-C (n = 8)	*p* value
Number of myelinated fibers of the right RLNs	240.3 ± 19.46	235.6 ± 8.77	0.54
Number of left myelinated fibers of the left RLNs	255.7 ± 31.96	307.8 ± 21.95	0.091
Diameter of myelinated axons in the right RLNs (μm)	4.89 ± 0.15	5.08 ± 0.25	0.82
Diameter of myelinated axons in the left RLNs (μm)	2.47 ± 0.09	2.72 ± 0.10	0.19

*p* < 0.05 versus PGA-C control (Student’s *t* test). Data are presented as mean ± SEM.

### Combination therapy of TrkA inhibitor and PGA-C tube prevented laryngeal muscle atrophy

To validate the effectiveness of TA muscle atrophy prevention, we performed a histological assessment of the harvested larynx specimens at 15 weeks after the surgery^25,28^.

**Figure 4:**
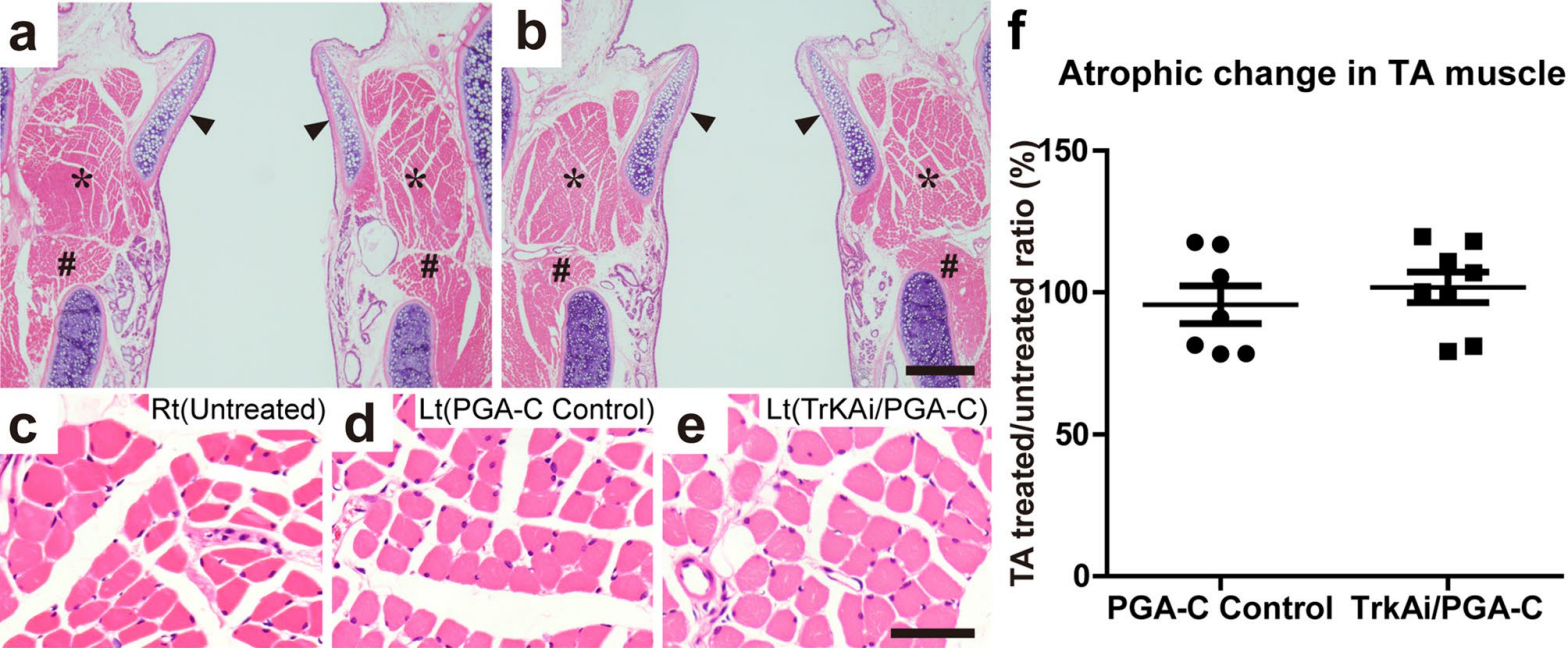
Assessment of thyroarytenoid (TA) muscle atrophy in PGA-C Control and TrkAi/PGA-C. (**a**,**b**) TA muscles in representative longitudinal sections stained with H&E. Scale bar is 500 µm. Arrowhead: arytenoid cartilage, *, TA muscle; #, lateral cricoarytenoid muscle. (**a**) PGA-C control. (**b**) TrkAi/PGA-C. (**c**–**e**) Images of TA muscles at a higher magnification. Scale bar is 50 µm. (**c**) Right TA muscle (untreated-control side). (**d**) Left TA (treated side) in PGA-C control. (**e**) Left TA (treated side) in TrkAi/PGA-C. Apparent atrophy of TA muscles was not observed between the treated and untreated sides in both groups. (**f**) The quantitative analysis did not show significant atrophic changes in treated/untreated ratio (%) of the area of the entire TA muscles. ns = *p* > 0.05.

In the larynx longitudinal sections stained with H&E, the left TA muscle fibers in the PGA-C Control and TrkAi/PGA-C were as thick as the right TA muscle fibers (Fig. 4a–e).

The treated/untreated ratios for the areas of the entire TA muscles that were extracted with ImageJ software (National Institutes of Health, Bethesda, Maryland, USA) were not different in the two groups (PGA-C Control (n = 7): 95.67% ± 6.63%, TrkAi/PGA-C (N = 8): 101.83% ± 5.41%, *p* = 0.48) (Fig. 4f). Thus, TA muscle atrophy was prevented in both groups.

### TrkA inhibitor prevented motor-sensory fiber misdirected regeneration

**Figure 5:**
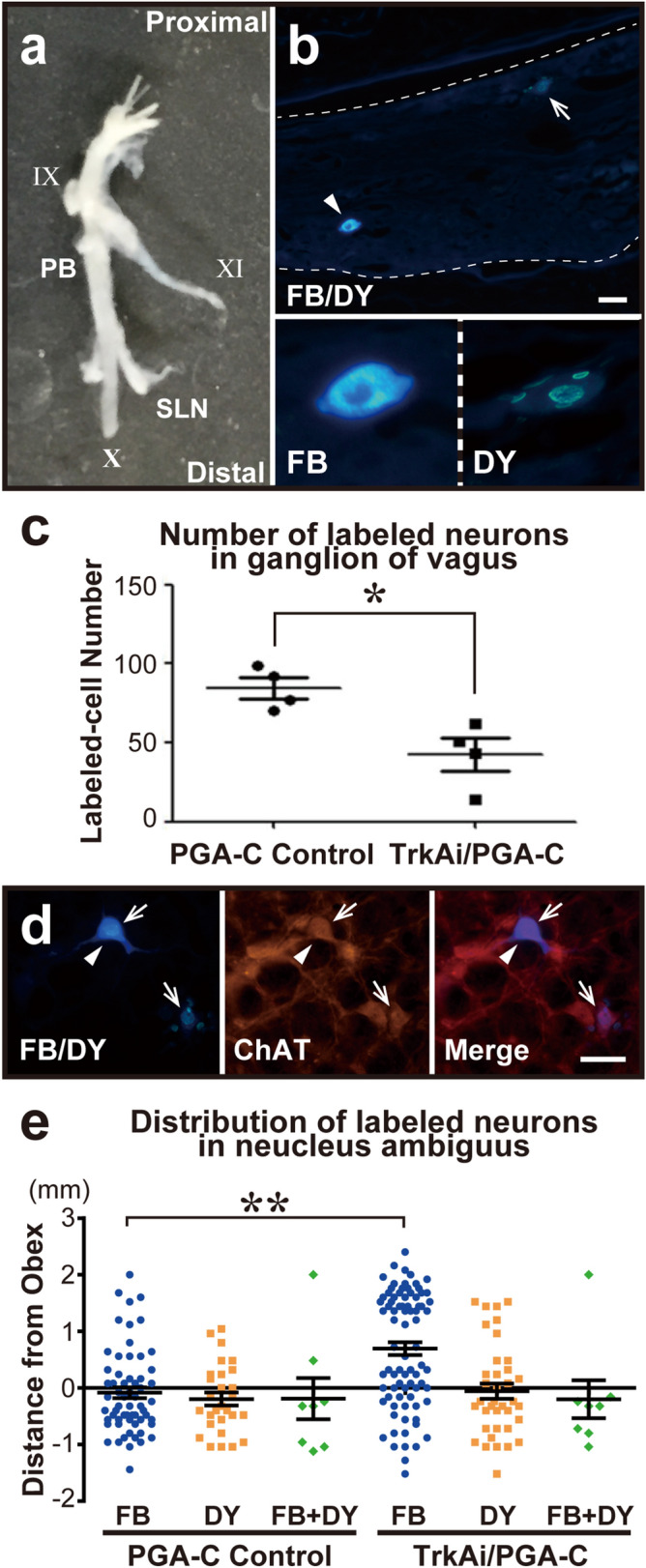
Assessment of retrograde labeling in ganglions of the vagus and nucleus ambiguus. (**a**) Harvested left ganglion of the vagus. PB: pharyngeal branch, SL: superior laryngeal nerve, IX: glossopharyngeal nerve, X: main trunk of the vagus nerve, XI: spinal accessory nerve. (**b**) Neurons in the ganglion of the vagus (surrounded by two broken lines) labeled with Fast Blue (FB) (arrowhead) and diamidino yellow dihydrochloride (DY) (arrow). The scale bar is 50 µm. (Below B) FB dye labeled the cytoplasm blue. DY dye labeled the nucleus yellow. (**c**) Comparison of the number of labeled neurons in the ganglion of the vagus. Labeled cell number had significantly decreased in TrkAi/PGA-C. *p < 0.05. (**d**) Triple-labeled neurons with FB, DY (FB/DY), and for ChAT immunofluorescence in the nucleus ambiguus. The arrows indicate DY labeled nuclei. The arrowheads indicate an FB labeled cytoplasm. In the ChAT-positive neurons, FB and/or DY labeled neurons (Merge) were counted. The scale bar is 20 µm. (**e**) The rostrocaudal distribution of labeled neurons in the nucleus ambiguus. The distribution of FB (injected into PCA muscles) labeled motor neurons from the obex had significantly shifted to a rostral location in TrkAi/PGA-C. There was no significant difference in the distribution of DY and FB + DY labeled neurons. FB + DY: FB and DY double-labeled neurons, **p < 0.01.

For assessment of misdirected regeneration involving sensory and motor fibers, at 14 weeks after surgery, we injected retrograde tracers, DY in the left TA muscles and FB in the left PCA muscles^11^. At one week after the injection, we harvested the left ganglions of the vagus, which contain primary sensory neurons of the RLN^29^ (Fig. 5a–c). The numbers of labeled neurons following injections to PCA and TA muscles were significantly different between the two groups (PGA-C Control (n = 4): 84.50 ± 6.67, TrkAi/PGA-C (n = 4): 42.50 ± 10.2, *p* = 0.029) (Fig. 5c).

The TrkA inhibitor, which induced a reduction in the labeled sensory neurons in the vagus ganglion, might have inhibited the misdirected regeneration of sensory nerve fibers to the TA and PCA.

### The distribution of labeled motor neurons in the nucleus ambiguus was shifted by TrkA inhibitor

To assess the post-procedure somatotopic changes in the nucleus ambiguus, we also harvested the brain tissue and determined the distribution of retrograde labeling with FB and DY dyes. In the same sections, we also performed immunofluorescence staining for ChAT to identify motor neurons (Fig. 5d).

The number of total labeled motor neurons in the nucleus ambiguus showed no significant difference between PGA-C Control and TrkAi/PGA-C (PGA-C Control (n = 4): 24.25 ± 9.11, TrkAi/PGA-C (n = 5): 25.20 ± 5.49, *p* = 0.93). The distribution of FB labeled motor neurons from the obex significantly shifted to a rostral location in TrkAi/PGA-C (PGA-C Control: − 85.25 ± 95.21, TrkAi/PGA-C: 693.83 ± 113.00, *p* < 0.0001) (Fig. 5e). More FB labeled motor neurons were observed near a location of 1,500 µm in TrkAi/PGA-C. Thus, the TrkA inhibitor did not increase the number of regenerated motor neurons, but it might have contributed to the recovery of vocal fold movement by rostral changing of distribution of FB labeled neurons and promoting regeneration of the PCA muscles, the sole abductor laryngeal muscle.

## Discussion

In this study, with the aim of improving misdirected regeneration among motor and sensory fibers, we investigated whether TrkA inhibitors could improve vocal fold function as a basis for PGA-Collagen tube treatment in an RLN axotomy model. Our study demonstrated a relatively high rate of motor function recovery of RLN, significant improvement of nerve fibers and CMAP, and prevention of vocal fold atrophy. Moreover, retrograde labeling showed that misdirected regeneration among motor and sensory fibers was inhibited and the distribution of FB (injected into PCA) labeled neurons in the nucleus ambiguus had shifted to the rostral side. These results may contribute to vocal fold motor function recovery.

The TrkA inhibitor that we used in this study blocks TrkA ATP binding sites with a high level of selectivity^30^. As stated above, peripheral nerve regeneration occurs as a result of binding between neurotrophic factors and their receptors, high-affinity Trk family receptors and low-affinity p75NTR. NGF mediates TrkA, affecting thin non-myelinated nociceptive receptor fibers (C fibers) and postganglionic automatic fibers^24,31^. BDNF and NT4/5 mediate TrkB to affect the thick myelinated tactile, pressure and vibration-sensing fibres (II fibers), and motor fibres (Aα fibers)^32,33^. NT-3 mediates TrkC to affect thick myelinated afferent fibers (Ia fibers, Ib fibers) running from the musculoskeletal system^34^. Meanwhile, p75NTR also binds with any of these four neurotrophic factors to be involved in pathways such as regulating cell death and cell survival of various types of nerves and controlling neurite extension^22,23^.

Upon binding NGF, TrkA dimerizes and auto-phosphorylates the tyrosine residues at the cytoplasmic tails. The selective blockade of TrkA phosphorylation inhibits downstream reactions through the extracellular signal-regulated kinase, phosphatidylinositol 3-kinase (PI3K), and phospholipase Cγ (PLC-γ) pathways^22^. This process may have selectively inhibited the regeneration of thin, non-myelinated nociceptive receptor fibers and postganglionic automatic fibers^24,35,36^ (Supplementary Fig. 1). In this study, we used this drug by having it permeate the PGA-Collagen tube at the transected site, thereby blocking sensory fiber regeneration within the tube and reducing misdirected regeneration among motor and sensory fibers.

Morphological evaluation of nerve fibers revealed more regenerated myelinated fibers in both groups than in the normal control group. In the peripheral nerves of TrkA knockout mice, almost all non-myelinated C fibers and approximately 50% of myelinated fibers were found to be reduced, and the affected myelinated fibers were thin, with a diameter ranging from 2–5 μm (thick myelinated fibers were not affected)^37^. The Trk tyrosine kinase inhibitor, K252a, did not block the neuritogenic effects of Schwann cells^38^. Conversely, p75NTR − / − mice exhibit reduced myelination with fewer myelinated axons and thinner myelin sheaths compared with normal mice. NGF-TrkA signaling does not appear to negatively affect Schwann cells that promote myelination^20,39–41^. However, this study does not focus on Schwann cells and other non-neuronal cells, therefore, its impact on other non-neuronal cells, including Schwann cells, is for future studies.

Our findings also indicated that the number of thick myelinated fibers was higher in the TrkAi/PGA-C (Fig. 3g), suggesting that TrkA inhibitors blocked the regeneration of thin myelinated or non-myelinated sensory fibers while thick myelinated nerve fibers were not affected, resulting in an increased proportion of motor nerve fibers, which are thick, myelinated fibers.

The electrophysiological evaluation indicated no significant improvements in MNCV in the TrkAi/PGA-C (Fig. 2c). To increase nerve conduction velocity, sufficient myelination, an insulating material, as well as an associated construction of node of Ranvier is required to promote the regeneration of thicker axons and saltatory conduction^42^. While the maximum axonal diameter improved to 6 μm in both groups, there was insufficient myelination compared to that on the normal side (Fig. 3a, d) in both groups (Fig. 3g). Thus, the improvement of myelinated fibers was not enough to cause saltatory conduction.

Meanwhile, CMAP improvement was noted in the TrkA inhibitor group (Fig. 2b, d). CMAP is evaluated by means of an M wave arising from muscle contraction due to the direct stimulation of alpha motor fibers or the H wave known as the Hoffman reflex determined by the motor neuron excitation after Ia afferent activation^43^. The increase in thick fibers in the TrkAi/PGA-C on fiber diameter histograms (Fig. 3g) appears to represent increases in alpha motor nerve fibers and Ia fibers (thick myelinated muscle afferent fibers), the regeneration of which was not blocked by TrkA inhibitors. Accordingly, the CMAP improvement appears to result from the increased alpha motor nerve fibers and Ia fibers (muscle afferent fibers) effectively binding with target muscles. Since a positive correlation between motor function recovery and CMAP recovery has been reported previously^44^, this result is consistent with our finding that motor function improved.

The motor neurons of the glottic adductors (TA) and abductors (PCA) are located in the nucleus ambiguus, while the primary sensory neurons are found in the vagus ganglion. Retrograde labeling has previously revealed that PCA-controlling neurons and TA-controlling neurons were distributed in the rostral and caudal areas, respectively, with some overlapping areas^45,46^. Previous studies have evaluated misdirected regeneration in RLN axotomy models by using retrograde labeling of motor neurons in the nucleus ambiguus^11,47^. Using the same method in the present study, we injected different retrograde tracers into the abductor and adductor laryngeal muscles in order to evaluate motor neuron distribution in the nucleus ambiguus. In addition, we evaluated for the first time labeled cells in the vagus ganglion after RLN injury (Fig. 5a–c). The labeled cells in the vagus ganglion were fewer in the TrkAi/PGA-C (Fig. 5c). We surmised that TrkA inhibitors suppressed the regeneration of sensory fibers, thereby decreasing the amount of misdirected regeneration among motor and sensory fibers. While no significant increases in labeled cell counts were noted in the nucleus ambiguus, distribution of FB labeled neurons was found to shift to the area around 1,500 µm rostral from the obex, where PCA-controlling neurons are dominantly located (Fig. 5e). The rostral shift of distribution of the FB labeled neurons controlling the PCA muscle, the sole abductor muscle, may have led to the improved motor functions of the vocal fold.

In this study, we found that blocking the NGF-TrkA pathway associated with the regeneration of non-myelinated C fibers, which are sensory fibers, in the TrkAi/PGA-C, thereby relatively promoted the regeneration of alpha motor fibers, myelinated Ia and Ib sensory fibers, which are involved in motor function. PCA motor nerve regeneration was also promoted with this, resulting in effective binding of laryngeal muscles to neuromuscular binding sites and enabling the achievement of good motor function improvement.

Drugs acting on the NGF-TrkA pathway have recently started to be clinically applied in the treatment of pain and cancer. Tanezumab, an NGF inhibitor, has been approved by the FDA as a first-track drug for chronic pain accompanying osteoarthropathy and chronic lower back pain^48^. The development of antineoplastic agents targeting the NGF-TrkA pathway is also underway^49^. Thus, the clinical application of drugs targeting TrkA appears likely in the near future.

The PGA-C tube (Nerbridge™) is clinically used in Japan and the US as a neural regeneration tube. As the collagen used in PGA-C tubes has been reported to show slow-release properties as in drug-delivery systems^50^, we have conducted additional experiments both in vitro and in vivo to clarify the quantity and time-course of release of the TrkA inhibitor (TrkAi) from the PGA-C tube as a scaffold. In vitro, the tube was filled with TrkAi soaked in 50 μl PBS, and the concentration of TrkAi leaking into the PBS was measured at various time points (0 days, 1 days, 3 days, 7 days, and 14 days). HPLC analysis was performed at each point. The sustained release effect of PGA-C tubes was observed for over 7 days (Supplementary Fig. 2a–d). In vivo, western blot analysis on the vagus ganglion in which TrkAi acted retrogradely showed that p-TrkA expression in the vagal ganglion was suppressed for more than 1 week (Supplementary Fig. 3a–c). These devices could be utilized as a basis for pharmacotherapy or gene therapy. The effects on other non-neuronal cells remain to be elucidated, but the local administration of TrkA inhibitors soaked in this tube did not cause any adverse reactions. This indicated that it is possible to apply our method clinically going forward. While TrkA inhibitors were not found to inhibit misdirected regeneration among motor fibers controlling the abductors and adductors, our results did exceed our expectations. Meanwhile, since paradoxical vocal fold movement was observed in one rat, the treatment of misdirected regeneration among motor neurons appears to be a task for the future.

The excessive axonal sprouting could, at least in part, be due to increased expression of trophic molecules at the lesion site. Accordingly, inhibition or blockade of these factors would reduce sprouting and improve the accuracy of reinnervation^41^. Streppel et al.^51^ reported that antibody-therapy for neurotrophic factors might reduce the branching of transected axons in facial nerve transected model. In terms of strategies for recovery of vocal fold mobility, Hernandez et al. reported that the timing of glial cell derived neurotrophic factor (GDNF) expression differs among the PCA and glottic adductors and abductors (LTA, MTA) following recurrent laryngeal nerve injury, and that this difference was associated with the order of immunohistological neuromuscular binding. They reported that regeneration first occurs in the PCA, and that the administration of anti-GDNF antibodies into the PCA, which is the sole abductor laryngeal muscle, blocks regeneration to the abductors, thereby promoting adductors regeneration and improving vocal fold motor function^52^. Thus, selectively promoting the regeneration of only the glottis abductors or adductors could enable some, although not complete, recovery in vocal fold motor function. It is suggested that differences in expression of neurotrophic factor in local and time course may contribute to selective regeneration and functional recovery.

In the present study, we found that treatment with TrkA inhibitors alone resulted in inhibition of misdirected regeneration among motor and sensory fibres, with a confirmed improvement rate of 50%. The electromyography results revealed respiratory synchronization in two rats in the TrkAi/PGA-C group (Fig. 2f). While TrkA inhibitors were not found to inhibit misdirected regeneration among motor fibers controlling the abductors and adductors, our results did exceed our expectations.

In conclusion, we investigated the function regenerative effects achieved by relative promotion of regeneration among motor fibers via prevention of regeneration among motor and sensory fibers. The results suggested that, in addition to promoting myelinated fibers regeneration and preventing laryngeal muscle atrophy, this approach also inhibited sensory nerve deviation into the laryngeal muscles by preventing the regeneration of sensory nerves and assisted the regeneration of motor neurons in PCA-controlling areas in the nucleus ambiguus. As a result, high rates of vocal fold movement recovery were achieved. While the issue of misdirected regeneration among motor neurons needs to be addressed as a future task, the treatment with PGA-C tube infiltrated in TrkA inhibitors has a strong potential for the clinical application.

## Materials and methods

### Animals

All experimental protocols for animal care, handling and experimentation was approved (Approval Numbers: 14069 and 2012–24) by the Ethics Committee of Animal Experiments of the National Defense Medical College (Tokorozawa, Saitama, Japan). Thirty-one adult male Sprague–Dawley rats (weighing over 400 g) were used in this study. In accordance with relevant guidelines and regulations, all procedures were performed under general anesthesia by intraperitoneal injections of ketamine hydrochloride (75 mg/kg) and xylazine hydrochloride (10 mg/kg), and all efforts were made to minimize pain and suffering.

### Surgical procedure

Animals were randomized into two groups as follows:

1. PGA-Collagen tube control group (PGA-C Control, n = 15),
2. TrkA inhibitor-infiltrated PGA-C tube treatment group (TrkAi/PGA-C, n = 16).

The surgical procedure was performed in two stages, with tracheostomy performed in each stage. The first surgical procedures were performed as reported previously^25,53^. Briefly, in the PGA-C Control, a PGA-Collagen Tube (Tube size; length: 3 mm, diameter: 0.5 mm) (Nerbridge™, Toyobo Co., Ltd., Osaka, Japan) was immersed in saline for 30–60 min before the operation. The RLN cut ends were bridged with the prepared PGA-Collagen tube with a 1-mm gap and then sutured to the tube with 10–0 nylon sutures as previously reported^25^. In the TrkAi/PGA-C treatment group, a PGA-Collagen tube was immersed in TrkA inhibitor (Cat. No. 648450, CALBIOCHEM, Darmstadt, Germany) at a concentration of IC 50 (63 nM against TrkA) for 30–60 min and the RLN cut ends were bridged in the same way. In both groups, the procedure site was covered and did not interfere with the tracheostomy orifice.

Each group was subdivided randomly into two subgroups as follows the first surgery:

- subgroup A: electrophysiological, morphological, and histological assessment subgroup (n = 7 [PGA-C Control] and n = 8 [TrkAi/PGA-C]).
- subgroup B: retrograde labeling subgroup for assessment of somatotopic changes in the nucleus ambiguus with motor neurons of the RLN and ganglion of the vagus with primary sensory neurons of the RLN (n = 8 [PGA-C Control] and n = 8 [TrkAi/PGA-C]).

Fourteen weeks after the first surgery, the second surgery for retrograde labeling was performed in subgroup B. Retrograde labeling on the thyroarytenoid (TA) muscle with diamidino yellow dihydrochloride (DY) (Sigma-Aldrich, D0281, CAS-No. 87397-07-7) and posterior cricoarytenoid (PCA) muscle with Fast Blue (FB) (Polysciences, Inc., Cat# 17740, CAS-No. 74749-42-1) was performed using a previously reported method^11^. FB or DY crystals were inserted with a 29-gauge needle. Excess dye was swabbed away following insertion of crystals to decrease contamination of other structures.

### Functional assessment

Fourteen weeks after the procedure, in both groups, vocal fold motion was evaluated and recorded with a laryngoscope (BRtra-1A; Bioresearch, Tokyo) and the LED Stella Scope (FS-S60; Daiichi Medical, Tokyo) attached to an iPhone 6 (Apple, Cupertino, CA).

Vocal fold movement was assessed by determining the left arytenoid motion angles in the CLOSE (adducted position) and OPEN phases (abducted position). A horizontal baseline was set at the intersection of the extended lines of both arytenoids of vocal folds. The angles between this baseline and the arytenoid line were defined as “α” for the adducted position and as “β” for the abducted position (Fig. 1a). Each angle was calculated using ImageJ software (NIH). Arytenoid motion angles were also defined as “α-β,” and their angle values were compared between each group^26,54,55^ (Fig. 1c).

### Electrophysiological analysis

Fifteen weeks after the procedure, in subgroup A, the RLN and PCA muscle were exposed on each side in the rats under general anesthesia. Electromyographic (EMG) analysis of the PCA muscle was conducted with a PowerLab computer-assisted EMG machine (AD Instruments Inc., Colorado Springs, CO) as reported previously^17^. Recording right angle electrodes were inserted into the PCA muscles. Compound muscle action potential (CMAP) and motor nerve conduction velocity (MNCV) of the right RLN (control side) and left RLN (treatment side) were measured under the stimulation of RLN (Fig. 2a–d). The maximum MNCVs were calculated based on the derived latencies and the distance between the two stimulating points (10 mm) with the following formula: MNCV (m/s) = 0.01 (m) × 1,000/[distal stimulation-derived action potential time (ms)–proximal stimulation-derived action potential time (ms)]. Additionally, without stimulation of RLN, resting potentials of both right and left PCA muscles, which contract in synchronization with breathing, were recorded at the same time (Fig. 2e, f).

### Histological study

After all procedures were performed within the 15-week period, the rats were perfused transcardially with 4% PFA in 0.1 M phosphate buffer (PB) (pH 7.4). Bilateral RLNs, larynges^25^, brains, and affected ganglions of the vagus nerve^29^ were dissected and post-fixed using the same method as that described in previous reports.

### Nerve regeneration

For electron microscopic study, the RLNs obtained from sites 5 mm distal and proximal to the transected site (next to tracheal ring 8) were fixed and handled as previously reported^27^. The 60-nm RLN sections were stained with uranyl acetate and citrate and examined by electron microscopy to evaluate axon regeneration. The number and diameter of the myelinated axons were measured with ImageJ software (NIH). The cross-sections of the whole RLN 5 mm distal from the treatment site were captured (Fig. 3a–f). The regenerated RLNs were evaluated by comparing the number of myelinated fibers and the diameter of myelinated axons. The diameters of myelinated axons were compared by distribution in each group (Fig. 3g).

### Laryngeal muscle atrophy

To assess laryngeal muscle atrophy, we observed TA muscles as we had reported previously^25^. Briefly, the dissected larynges were sliced (5 µm) into longitudinal sections (corresponding to the coronal plane in human) and stained with hematoxylin and eosin (H&E). TA muscle atrophy was evaluated by comparing the areas of the treated (left) and untreated (right) sides (treated/untreated ratio) in each animal with ImageJ software (NIH), and the ratio was averaged in each group as described previously^25,28^.

### Retrograde labeling

In this study, we used FB and DY as retrograde tracers. Fluorescent-labeled neurons were visualized with a confocal microscope (LSM 510; Carl Zeiss, Jena, Germany) with fluorescence activation for both FB and DY (excitation wavelength, 355–425 µm).

### Retrograde labeling in ganglions of the vagus

For assessment of misdirected regeneration between sensory and motor fibers, the left ganglions of the vagus, which are the primary sensory neurons of the RLN, were dissected as previously reported^29^. The ganglion specimens were cryoprotected in 30% glucose and frozen on dry ice. Serial frozen sections 6 µm thick were obtained with a Microm HM560 cryostat (Thermo scientific, Kalamazoo, MI) and observed to count the labeled neurons.

### Retrograde labeling in the nucleus ambiguus

For assessment of somatotopic changes in motor neurons in the nucleus ambiguus, the brains were dissected and cryoprotected in 20% glycerin in PB and frozen on dry ice. Serial coronal frozen sections (40 µm thick) were obtained by using a sliding microtome (HM440E; Thermo scientific) and separated into 4 series^56^. We used series 1 and 3 to avoid double-counting.

In the brain specimens from pyramidal decussation to facial nucleus, two series of sections were processed for immunofluorescence staining for anti-choline acetyltransferase (ChAT) to elucidate whether the FB and/or DY (FB/DY)-labelled neurons were motor neurons. In brief, the sections were blocked with 25% BlockAce in phosphate-buffered saline (PBS) (pH 7.4) containing 0.5% Triton-X and incubated in goat anti-ChAT antibody (1:200; Merck Millipore, Billerica, MA). Then, the sections were washed in PBS and incubated in Cy3-conjugated anti-goat IgG (1:400; Jackson ImmunoResearch, West Grove, PA). After washing, the sections were mounted, dried, and cover-slipped with DPX. Motor neurons of the nucleus ambiguus were identified based on ChAT immunoreactivity and the location in the reticular formation, and double-labelled (FB/DY and ChAT) neurons in the nucleus ambiguus were counted (Fig. 5d). As for the somatotopic changes, a distribution chart was created centering on the obex from every other section.

### Statistical analysis

Statistical analyses were performed using Prism 5 (GraphPad Software, Inc., La 241 Jolla, CA, USA). All results are expressed as the mean ± SEM. Unpaired two-tailed Student’s *t* test was used to compare arytenoid motion angle data, electrophysiological data, nerve morphological data, laryngeal muscle histological data and retrograde labeling data between the two groups. The Chi-squared test was used to compare the mobility of vocal cord movement between the two groups. Odds ratios were considered statistically significant when the lower limit of 95% CI exceeded 1.0. A *p* < 0.05 was considered statistically significant.

## Supplementary information

Supplementary Data.

Supplementary Figure 1.

Supplementary Figure 2.

Supplementary Figure 3.

Supplementary Figure 4.

Supplementary Information.

Supplementary Video 2.

Supplementary Video 1.

Supplementary Video Legend.

## Data Availability

Data underlying Figs. 1, 2, 3, 4 and 5 and Supplementary Figs. 2 and 3, are provided as a Source Data file. Other data are available from the corresponding authors upon a reasonable request.
