# Supplementary material for: TrkA inhibitor promotes motor functional regeneration of recurrent laryngeal nerve by suppression of sensory nerve regeneration
Source: Sci Rep. 2020 Oct 9;10:16892. doi: 10.1038/s41598-020-72288-w (PMC7547101; doi:10.1038/s41598-020-72288-w)
Supplement: Supplementary file 1 — Supplementary Data. [file 41598_2020_72288_MOESM1_ESM.docx]

**Fig.1c**

|  | PGA-C Control | | | | | |  |  | TrkAi/PGA-C | | | | | |
| --- | --- | --- | --- | --- | --- | --- | --- | --- | --- | --- | --- | --- | --- | --- |
|  | Right | | | Left | | |  |  | Right | | | Left | | |
| Rat No. | α | β | Angle (α-β) | α | β | Angle (α-β) |  | Rat No. | α | β | Angle (α-β) | α | β | Angle (α-β) |
| R1 | 81.966 | 78.318 | 3.648 | 79.864 | 79.864 | 0 |  | R16 | 89.84 | 81.934 | 7.906 | 74.62 | 74.62 | 0 |
| R2 | 80.764 | 76.349 | 4.415 | 83.634 | 81.549 | 2.085 |  | R17 | 60.558 | 54.67 | 5.888 | 103.259 | 103.259 | 0 |
| R3 | 87.61 | 85.796 | 1.814 | 85.03 | 85.03 | 0 |  | R18 | 92.49 | 86.372 | 6.118 | 70.739 | 64.775 | 5.964 |
| R4 | 88.095 | 85.8 | 2.295 | 78.12 | 78.12 | 0 |  | R19 | 81.012 | 75.122 | 5.89 | 85.162 | 81.716 | 3.446 |
| R5 | 75.247 | 70.813 | 4.434 | 77.047 | 77.047 | 0 |  | R20 | 87.061 | 81.661 | 5.4 | 72.94 | 72.933 | 0.007 |
| R6 | 83.242 | 79.177 | 4.065 | 85.914 | 85.914 | 0 |  | R21 | 87.274 | 81.956 | 5.318 | 76.562 | 76.562 | 0 |
| R7 | 83.618 | 79.678 | 3.94 | 82.964 | 82.558 | 0.406 |  | R22 | 85.373 | 81.216 | 4.157 | 86.252 | 83.871 | 2.381 |
| R8 | 81.242 | 77.039 | 4.203 | 90 | 90 | 0 |  | R23 | 78.166 | 71.769 | 6.397 | 85.903 | 82.643 | 3.26 |
| R9 | 59.073 | 48.275 | 10.798 | 97.408 | 97.408 | 0 |  | R24 | 88.152 | 80.772 | 7.38 | 86.221 | 80.981 | 5.24 |
| R10 | 83.63 | 81.151 | 2.479 | 86.295 | 85.475 | 0.82 |  | R25 | 83.054 | 79.291 | 3.763 | 87.385 | 87.385 | 0 |
| R11 | 104.725 | 93.036 | 11.689 | 67.101 | 66.199 | 0.902 |  | R26 | 81.611 | 77.63 | 3.981 | 77.542 | 74.557 | 2.985 |
| R12 | 80.263 | 71.381 | 8.882 | 80.67 | 80.134 | 0.536 |  | R27 | 73.171 | 56.162 | 17.009 | 71.565 | 64.809 | 6.756 |
| R13 | 85.715 | 82.497 | 3.218 | 82.046 | 81.641 | 0.405 |  | R28 | 84.404 | 78.545 | 5.859 | 87.131 | 86.947 | 0.184 |
| R14 | 74.451 | 71.322 | 3.129 | 70.787 | 70.787 | 0 |  | R29 | 85.655 | 81.064 | 4.591 | 79.665 | 79.665 | 0 |
| R15 | 89.948 | 86.353 | 3.595 | 76.819 | 76.819 | 0 |  | R30 | 98.101 | 93.034 | 5.067 | 69.444 | 66.61 | 2.834 |
|  |  |  |  |  |  |  |  | R31 | 76.896 | 73.149 | 3.747 | 90.822 | 90.822 | 0 |

**Fig.2c** MNCV

|  | PGA-C Control | |  |  | TrkAi/PGA-C | |
| --- | --- | --- | --- | --- | --- | --- |
| Rat No. | Right | Left |  | Rat No. | Right | Left |
| R2 | 25.641 | 52.63157 |  | R16 | 15.684 | 43.478 |
| R3 | 25 | 52.63157 |  | R17 | 52.632 | 66.666 |
| R4 | 19.608 | 62.5 |  | R18 | 17.857 | 71.428 |
| R5 | 33.3333 | 71.4285 |  | R19 | 14.286 | 76.923 |
| R6 | 33.3333 | 83.3333 |  | R21 | 23.81 | 50 |
| R7 | 24.3902 | 71.4285 |  | R22 | 35.714 | 90.909 |
|  |  |  |  | R23 | 38.4615 | 71.4285 |

**Fig.2d** CMAP

|  | PGA-C Control | | |  |  | TrkAi/PGA-C | | |
| --- | --- | --- | --- | --- | --- | --- | --- | --- |
| Rat No. | Right | Left | Ratio |  | Rat No. | Right | Left | Ratio |
| R2 | 1.787 | 1.248 | 69.837717 |  | R16 | 4.28 | 4.7036 | 109.8972 |
| R3 | 4.9425 | 0.23775 | 4.8103187 |  | R17 | 1.025 | 1.0007 | 97.629268 |
| R4 | 3.8144 | 2.5956 | 68.047399 |  | R18 | 1.9381 | 1.252 | 64.59935 |
| R5 | 3.8406 | 2.5963 | 67.601416 |  | R19 | 0.505 | 0.421 | 83.366337 |
| R6 | 1.8006 | 0.364 | 20.215484 |  | R21 | 3.0319 | 5.295 | 174.64296 |
| R7 | 7.4619 | 1.6043 | 21.499886 |  | R22 | 0.6956 | 0.4193 | 60.278896 |
|  |  |  |  |  | R23 | 9.0778 | 5.3081 | 58.473419 |

**Fig.3g** Histogram of Axon Diameters

| Number of Fibers |  |  |  |  |  |  |  |  |  |  |  |  |  |
| --- | --- | --- | --- | --- | --- | --- | --- | --- | --- | --- | --- | --- | --- |
| Diameters(μm) | 0～1 | 1～2 | 2～3 | 3～4 | 4～5 | 5～6 | 6～7 | 7～8 | 8～9 | 9～10 | 10～11 | 11～12 | 12～ |
| Untreated-Control | 37 | 472 | 763 | 395 | 421 | 493 | 526 | 328 | 186 | 133 | 60 | 21 | 13 |
| PGA-C control | 9 | 734 | 644 | 227 | 106 | 54 | 10 | 5 | 0 | 1 | 0 | 0 | 0 |
| TrkAi/PGA-C | 11 | 769 | 847 | 441 | 254 | 98 | 23 | 5 | 1 | 0 | 0 | 0 | 0 |
|  |  |  |  |  |  |  |  |  |  |  |  |  |  |
| Ditribution(%) |  |  |  |  |  |  |  |  |  |  |  |  |  |
| Diameters(μm) | 0～1 | 1～2 | 2～3 | 3～4 | 4～5 | 5～6 | 6～7 | 7～8 | 8～9 | 9～10 | 10～11 | 11～12 | 12～ |
| Untreated-Control | 0.96153 | 12.2661 | 19.8284 | 10.2650 | 10.9407 | 12.8118 | 13.6694 | 8.52390 | 4.83367 | 3.45634 | 1.55925 | 0.5457 | 0.33783 |
| PGA-C control | 0.50279 | 41.0055 | 35.9776 | 12.6815 | 5.92178 | 3.01675 | 0.55865 | 0.27932 | 0 | 0.05586 | 0 | 0 | 0 |
| TrkAi/PGA-C | 0.44916 | 31.4005 | 34.5855 | 18.0073 | 10.3715 | 4.00163 | 0.93915 | 0.20416 | 0.04083 | 0 | 0 | 0 | 0 |

Fig.4f

| TA Area |  |  |  |  |  |  |  |  |
| --- | --- | --- | --- | --- | --- | --- | --- | --- |
|  | PGA-C Control | | |  |  | TrkAi/PGA-C | | |
| Rat No. | Right | Left | Rtio |  | Rat No. | Right | Left | Rtio |
| R1 | 227928 | 207667 | 91.110789 |  | R16 | 176166 | 139665 | 79.280338 |
| R2 | 186168 | 151831 | 81.555906 |  | R17 | 251315 | 204037 | 81.187752 |
| R3 | 149597 | 117269 | 78.389941 |  | R18 | 226408 | 242128 | 106.94322 |
| R4 | 217236 | 255782 | 117.74384 |  | R19 | 163329 | 192739 | 118.0066 |
| R5 | 201251 | 212146 | 105.41364 |  | R20 | 212502 | 209254 | 98.471544 |
| R6 | 178784 | 140405 | 78.533314 |  | R21 | 174122 | 208371 | 119.66954 |
| R7 | 204371 | 239063 | 116.97501 |  | R22 | 181156 | 200883 | 110.88951 |
|  |  |  |  |  | R23 | 168556 | 168840 | 100.16849 |

**Fig.5c**

| Number of labeled neurons in ganglion of vagus | | | |
| --- | --- | --- | --- |
| PGA-C Control | | TrkAi/PGA-C | |
| R9 | 70 | R25 | 43 |
| R10 | 92 | R26 | 14 |
| R11 | 99 | R29 | 62 |
| R13 | 77 | R30 | 51 |

**Fig.5e**

| Distance from Obex | |  |  |  |  |
| --- | --- | --- | --- | --- | --- |
| PGA-C Control | | | TrkAi/PGA-C | | |
| FB | DY | FB+DY | FB | DY | FB+DY |
| -960 | -160 | -1040 | 240 | -1520 | 2000 |
| -560 | -1040 | -240 | 320 | -1040 | -1040 |
| -480 | -1040 | -1120 | 320 | -1040 | -720 |
| -480 | -1040 | -960 | 400 | -960 | -240 |
| -400 | -960 | -320 | 560 | -720 | -800 |
| -400 | -880 | -320 | 1600 | -720 | -320 |
| 80 | -560 | 2000 | 1920 | -720 | -320 |
| -1440 | -480 | 480 | 1960 | 0 | -160 |
| -1040 | -480 |  | 2000 | 320 |  |
| -960 | -400 |  | 2080 | -1040 |  |
| -880 | -400 |  | 2160 | -880 |  |
| -880 | -400 |  | 0 | -560 |  |
| -880 | -320 |  | 0 | -400 |  |
| -800 | 320 |  | 80 | -320 |  |
| -640 | 960 |  | 320 | 80 |  |
| -640 | 1040 |  | 320 | -240 |  |
| -640 | -640 |  | 1520 | 240 |  |
| -640 | -480 |  | -1520 | 480 |  |
| -560 | 0 |  | -1280 | 960 |  |
| -560 | 0 |  | -880 | 1200 |  |
| -560 | 240 |  | -800 | 1440 |  |
| -480 | -800 |  | -480 | 1440 |  |
| -480 | -160 |  | -320 | 1520 |  |
| -480 | 160 |  | 80 | 1520 |  |
| -480 | 240 |  | 80 | -960 |  |
| -400 | 480 |  | 240 | -400 |  |
| -320 | 480 |  | 1520 | -400 |  |
| -320 | 800 |  | -1040 | -320 |  |
| -320 |  |  | -1040 | -320 |  |
| 0 |  |  | -1040 | -320 |  |
| 160 |  |  | -880 | -240 |  |
| 560 |  |  | -800 | -240 |  |
| 560 |  |  | -640 | 80 |  |
| 640 |  |  | -560 | 160 |  |
| 640 |  |  | -560 | 240 |  |
| 1120 |  |  | 0 | 480 |  |
| -960 |  |  | 0 | 1120 |  |
| -560 |  |  | 720 |  |  |
| -240 |  |  | 720 |  |  |
| -160 |  |  | 1360 |  |  |
| -160 |  |  | -320 |  |  |
| -160 |  |  | -320 |  |  |
| -80 |  |  | -240 |  |  |
| -80 |  |  | -240 |  |  |
| 160 |  |  | -160 |  |  |
| 240 |  |  | -160 |  |  |
| 1680 |  |  | -160 |  |  |
| -800 |  |  | 0 |  |  |
| 80 |  |  | 240 |  |  |
| 80 |  |  | 1520 |  |  |
| 80 |  |  | 1600 |  |  |
| 80 |  |  | 1840 |  |  |
| 320 |  |  | 1840 |  |  |
| 320 |  |  | 1440 |  |  |
| 560 |  |  | 1440 |  |  |
| 800 |  |  | 1440 |  |  |
| 1600 |  |  | 1440 |  |  |
| 1200 |  |  | 1600 |  |  |
| 1200 |  |  | 1680 |  |  |
| 1520 |  |  | 1360 |  |  |
| 2000 |  |  | 1440 |  |  |
|  |  |  | 1680 |  |  |
|  |  |  | 1680 |  |  |
|  |  |  | 1680 |  |  |
|  |  |  | 240 |  |  |
|  |  |  | 1120 |  |  |
|  |  |  | 1200 |  |  |
|  |  |  | 1360 |  |  |
|  |  |  | 1360 |  |  |
|  |  |  | 1440 |  |  |
|  |  |  | 1200 |  |  |
|  |  |  | 1360 |  |  |
|  |  |  | 1360 |  |  |
|  |  |  | 1600 |  |  |
|  |  |  | 1680 |  |  |
|  |  |  | 1760 |  |  |
|  |  |  | 1760 |  |  |
|  |  |  | 1760 |  |  |
|  |  |  | 1760 |  |  |
|  |  |  | 1840 |  |  |
|  |  |  | 2400 |  |  |

**Supplementary Fig.2b**

| TrkAi denisty (ng/ml) | peak height | peak area |
| --- | --- | --- |
| 200 | 14610 | 92048 |
| 20 | 1159 | 7323 |

**Supplementary Fig.2c,d**

| Day | peak height | peak area | density | Cumulative density |
| --- | --- | --- | --- | --- |
| 0 | 13666 | 83587 | 182.025 | 182.025 |
| 1 | 12200 | 75076 | 163.942 | 345.967 |
| 3 | 6759 | 40861 | 91.2532 | 437.220 |
| 7 | 3377 | 20803 | 48.6378 | 485.858 |
| 14 | 1137 | 7291 | 19.93329 | 505.791 |

**Supplementary Fig.3b,c**

| 1 |  |  |  |  |  |
| --- | --- | --- | --- | --- | --- |
|  | Pre-Control | PGA-C Control 1W | PGA-C Control 2W | TrkAi/PGA-C 1W | TrkAi/PGA-C 2W |
| β-actin | 325 | 253 | 412 | 312 | 425 |
| TrkA | 125 | 102 | 112 | 97 | 145 |
| TrkA/β-actin | 0.384615385 | 0.403162055 | 0.27184466 | 0.310897436 | 0.341176471 |
| TrkA(norm) | 1 | 1.048221344 | 0.706796117 | 0.808333333 | 0.887058824 |
| pTrkA | 85 | 192 | 142 | 53 | 225 |
| pTrkA/β-actin | 0.261538462 | 0.758893281 | 0.344660194 | 0.169871795 | 0.529411765 |
| pTrkA(norm) | 1 | 2.901650779 | 1.317818389 | 0.649509804 | 2.024221453 |
| pTrkA/Trk(norm) | 1 | 2.76816609 | 1.864495798 | 0.803517283 | 2.281947262 |
|  |  |  |  |  |  |
| 2 |  |  |  |  |  |
|  | Pre-Control | PGA-C Control 1W | PGA-C Control 2W | TrkAi/PGA-C 1W | TrkAi/PGA-C 2W |
| β-actin | 286 | 259 | 305 | 278 | 321 |
| TrkA | 105 | 135 | 138 | 120 | 158 |
| TrkA/β-actin | 0.367132867 | 0.521235521 | 0.452459016 | 0.431654676 | 0.492211838 |
| TrkA(norm) | 1 | 1.419746277 | 1.232412178 | 1.175745118 | 1.340691292 |
| pTrkA | 52 | 129 | 125 | 75 | 145 |
| pTrkA/β-actin | 0.181818182 | 0.498069498 | 0.409836066 | 0.269784173 | 0.451713396 |
| pTrkA(norm) | 1 | 2.739382239 | 2.254098361 | 1.48381295 | 2.484423676 |
| pTrkA/Trk(norm) | 1 | 1.929487179 | 1.829013378 | 1.262019231 | 1.853091529 |
|  |  |  |  |  |  |
| 3 |  |  |  |  |  |
|  | Pre-Control | PGA-C Control 1W | PGA-C Control 2W | TrkAi/PGA-C 1W | TrkAi/PGA-C 2W |
| β-actin | 112 | 105 | 98 | 85 | 79 |
| TrkA | 205 | 187 | 222 | 178 | 154 |
| TrkA/β-actin | 1.830357143 | 1.780952381 | 2.265306122 | 2.094117647 | 1.949367089 |
| TrkA(norm) | 1 | 0.97300813 | 1.237630662 | 1.1441033 | 1.065020068 |
| pTrkA | 215 | 487 | 368 | 175 | 302 |
| pTrkA/β-actin | 1.919642857 | 4.638095238 | 3.755102041 | 2.058823529 | 3.82278481 |
| pTrkA(norm) | 1 | 2.416124031 | 1.956146179 | 1.07250342 | 1.99140418 |
| pTrkA/Trk(norm) | 1 | 2.483148862 | 1.580557301 | 0.937418343 | 1.869827847 |
|  |  |  |  |  |  |
|  |  |  |  |  |  |
|  |  |  |  |  |  |
|  |  |  |  |  |  |
|  |  |  |  |  |  |
|  |  |  |  |  |  |
|  |  |  |  |  |  |
| 4 |  |  |  |  |  |
|  | Pre-Control | PGA-C Control 1W | PGA-C Control 2W | TrkAi/PGA-C 1W | TrkAi/PGA-C 2W |
| β-actin | 273 | 260 | 286 | 263 | 257 |
| TrkA | 123 | 107 | 99 | 115 | 125 |
| TrkA/β-actin | 0.450549451 | 0.411538462 | 0.346153846 | 0.437262357 | 0.486381323 |
| TrkA(norm) | 1 | 0.913414634 | 0.768292683 | 0.970509135 | 1.079529278 |
| pTrkA | 336 | 385 | 285 | 65 | 342 |
| pTrkA/β-actin | 1.230769231 | 1.480769231 | 0.996503497 | 0.247148289 | 1.3307393 |
| pTrkA(norm) | 1 | 1.203125 | 0.809659091 | 0.200807985 | 1.081225681 |
| pTrkA/TrkA(norm) | 1 | 1.317172897 | 1.053841991 | 0.206909938 | 1.001571429 |
