# Supplementary material for: TrkA inhibitor promotes motor functional regeneration of recurrent laryngeal nerve by suppression of sensory nerve regeneration
Source: Sci Rep. 2020 Oct 9;10:16892. doi: 10.1038/s41598-020-72288-w (PMC7547101; doi:10.1038/s41598-020-72288-w)
Supplement: Supplementary file 6 — Supplementary Information. [file 41598_2020_72288_MOESM6_ESM.docx]

**Supplementary Information (SI)**

**TrkA inhibitor promotes motor functional regeneration of recurrent laryngeal nerve by suppression of sensory nerve regeneration**

Hiroshi Suzuki^1,2^, Koji Araki^1^, Toshiyasu Matsui^3,4^, Yuya Tanaka^1^, Kosuke Uno^1^, Masayuki Tomifuji^1^, Taku Yamashita^1,5^, Yasushi Satoh^6^, Yasushi Kobayashi^3^, Akihiro Shiotani^1^

^1^Department of Otolaryngology-Head and Neck Surgery, National Defense Medical College

^2^Department of Otolaryngology, Self-Defense Forces Central Hospital

^3^Department of Anatomy and Neurobiology, National Defense Medical College

^4^Laboratory of Veterinary Anatomy, Faculty of Veterinary Medicine, Okayama University of Science

^5^Department of Otolaryngology-Head and Neck Surgery, Kitasato University School of Medicine

^6^Department of Biochemistry, National Defense Medical College

Corresponding author:

Koji Araki, MD, PhD;

Associate Professor, Department of Otolaryngology-Head and Neck Surgery National Defense Medical College, 3-2 Namiki, Tokorozawa, Saitama 3598513, Japan

Phone: +81-4-2995-1686, Fax: +81-4-2996-5212

Email: [kojaraki@ndmc.ac.jp](mailto:kojaraki@ndmc.ac.jp)

**SI Materials and Methods**

**HPLC analysis for drug delivery release of PGA-C scaffolds**

The studies of drug delivery release of the PGA-C scaffolds were performed in PBS for 2 weeks and the UV absorbance of the solution was measured at various time points (0 d, 1 d, 3 d, 7 d, and 14 d) as previously reported [1,2]. In this study, we prepared 6 vials; 4 PGA-C tubes (diameter, 1 mm; length, 3 mm; approximately 2 µL) soaked in TrkAi (1000ng/ml) were used for each vial. For every measurement, 50 µL of solution was taken from the vial and replaced with fresh PBS. The peak of TrkAi absorbance spectrum was detected using a UV-1800 chromatography (SHIMADZU, Kyoto, Japan) (Supplemental Fig.2a). Each solution was collected at each point and analysed using a J-Pak Supero C18 column (4.6 × 150 mm×5 μm; Jasco Engineering, Tokyo, Japan) and connected by an Extrema HPLC system (Jasco, Tokyo, Japan). The analytical conditions for TrkAi are shown in Supplementary Table 1. The calibration curve calculated from the 20 ng/ml and 200 ng/ml data (Supplementary Figure 2b) was used. To determine the release profile of the scaffold, the drug release curves were plotted　(Supplementary Fig. 2c and d).

**Western blot analysis**

In order to confirm the duration of the inhibitory effect of TrkAi on sensory neurons *in vivo*, western blot analysis of protein lysate from the left vagus ganglion was performed as previously described [3]. Proteins were obtained from left vagus ganglions harvested at pre-operation (Pre), 1 week following PGA-C sham operation (PGA-C Control, 1 w), 1 week following TrkAi/PGA-C operation (TrkAi/PGA-C 1W), 2 weeks following PGA-C sham operation (PGA-C control, 2 w), and 2 weeks following TrkAi/PGA-C operation (TrkAi/PGA-C 1W) (n = 4 rats for each group). The homogenate proteins were subjected to SDS-PAGE. The proteins were transferred onto PVDF (polyvinylidene fluoride) membranes (Immobilon-P, Millipore, Bedford, MA) and the blots were immunoreacted with primary antibodies. The primary antibodies included anti-phospho-TrkA (p-TrkA) (abcam, ab1445), anti-TrkA (abcam, ab76291), and anti-β-actin (mouse monoclonal, Sigma-Aldrich, St. Louis, MO, USA). Secondary antibodies included horseradish peroxidase (HRP)-linked anti-rabbit immunoglobulin G (IgG) (#7074, goat polyclonal, Cell Signaling Technology) and HRP-linked anti-mouse IgG (#7076, horse polyclonal, Cell Signaling Technology). The protein bands were visualized by chemiluminescence detection system (Immunostar, Wako, Osaka, Japan) and blot images were captured on a luminescent image analyzer (Amersham Imager 600, GE Healthcare, Tokyo, Japan).

**SI Result**

**HPLC analysis revealed TrkAi sustained release of PGA-C scaffolds *in vitro***

The peak of TrkAi absorbance spectrum was 380 nm (Supplementary Fig. 2a).

The intensity peak decreased with time, but not sharply, and was detectable for over 7 days (Supplementary Fig. 2c and d). These results indicate that the inhibitor can be released sustainingly from the conduit for over 7 days.

**The TrkA inhibitor from PGA-C suppressed phosphorylation of TrkA in vagus ganglion at least for 1 week *in vivo***

Western blot analysis showed that the expression levels of TrkA were unchanged over time regardless of group (p > 0.05, one-way ANOVA, n = 4 rats for each) (Supplementary Fig.3a, b). The phosphorylation level of TrkA in the vagus ganglion in the PGA-C/TrkAi group at 1 week following the treatment was significantly decreased compared to that of the PGA-Collagen tube bridging control group (p < 0.01, one-way ANOVA, n = 4 rats for each, Supplementary Fig.3a, c). These results indicate that the inhibitor was effective *in vivo* at least for 1 week.

**References**

1 Hou, J. *et al.* Sustained release of N-acetylcysteine by sandwich structured polycaprolactone/collagen scaffolds for wound healing. *Journal of biomedical materials research. Part A* **107**, 1414-1424, doi:10.1002/jbm.a.36656 (2019).

2 Santos, D., Giudetti, G., Micera, S., Navarro, X. & Del Valle, J. Focal release of neurotrophic factors by biodegradable microspheres enhance motor and sensory axonal regeneration in vitro and in vivo. *Brain Res* **1636**, 93-106, doi:10.1016/j.brainres.2016.01.051 (2016).

3 Satoh, Y. *et al.* ERK2 contributes to the control of social behaviors in mice. *The Journal of neuroscience : the official journal of the Society for Neuroscience* **31**, 11953-11967, doi:10.1523/JNEUROSCI.2349-11.2011 (2011).

**Supplementary Data**

**
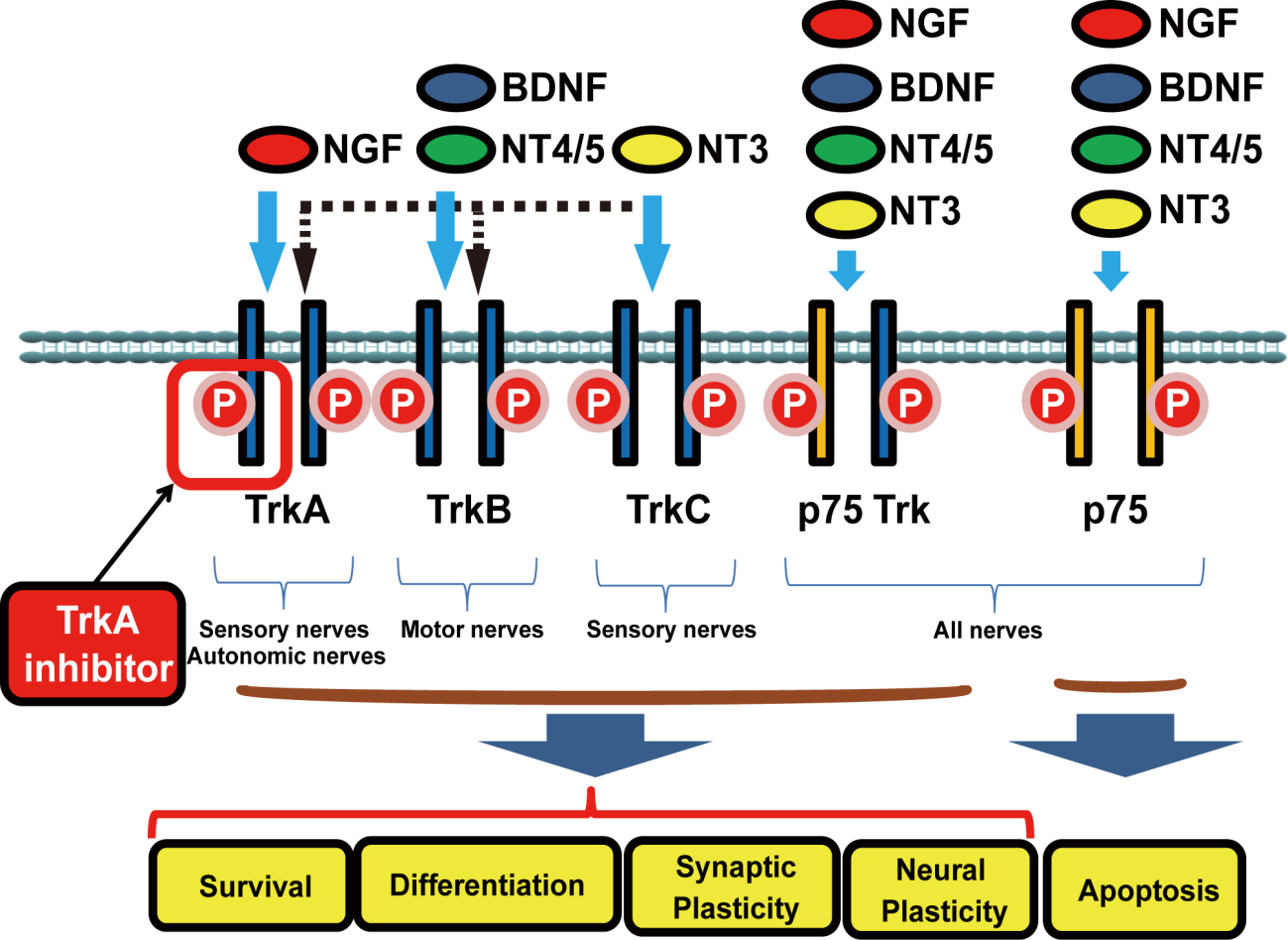
**

**Supplementary Figure 1.** **Overview of nerve regeneration pathways that are linked to each neurotrophic factor and each receptor.** The family of neurotrophins consists of four growth factors that bind to p75NTR (homodimer or heterodimer of p75) with low affinity and to their respective high-affinity tropomyosin-related kinase (Trk) receptors: NGF to TrkA, BDNF and NT-4/5 to TrkB, and NT-3 to TrkC (blue arrow). NT-3 can to some extent also bind and activate noncognate TrkA and B receptors (dotted arrow). Upon binding of neurotrophin, the cognate Trk is activated by tyrosine phosphorylation in the cytoplasmic domain and consequently recruits and activates downstream signaling molecules to trigger various physiological responses. The NGF-TrkA pathway is related to sensory and sympathetic nerve regeneration with activation of MAPK, PI3K, and PLC γ pathways mediated mainly by phosphorylation (red spheres). The TrkA inhibitor inhibits TrkA activation and its downregulation to nerve regeneration.

**
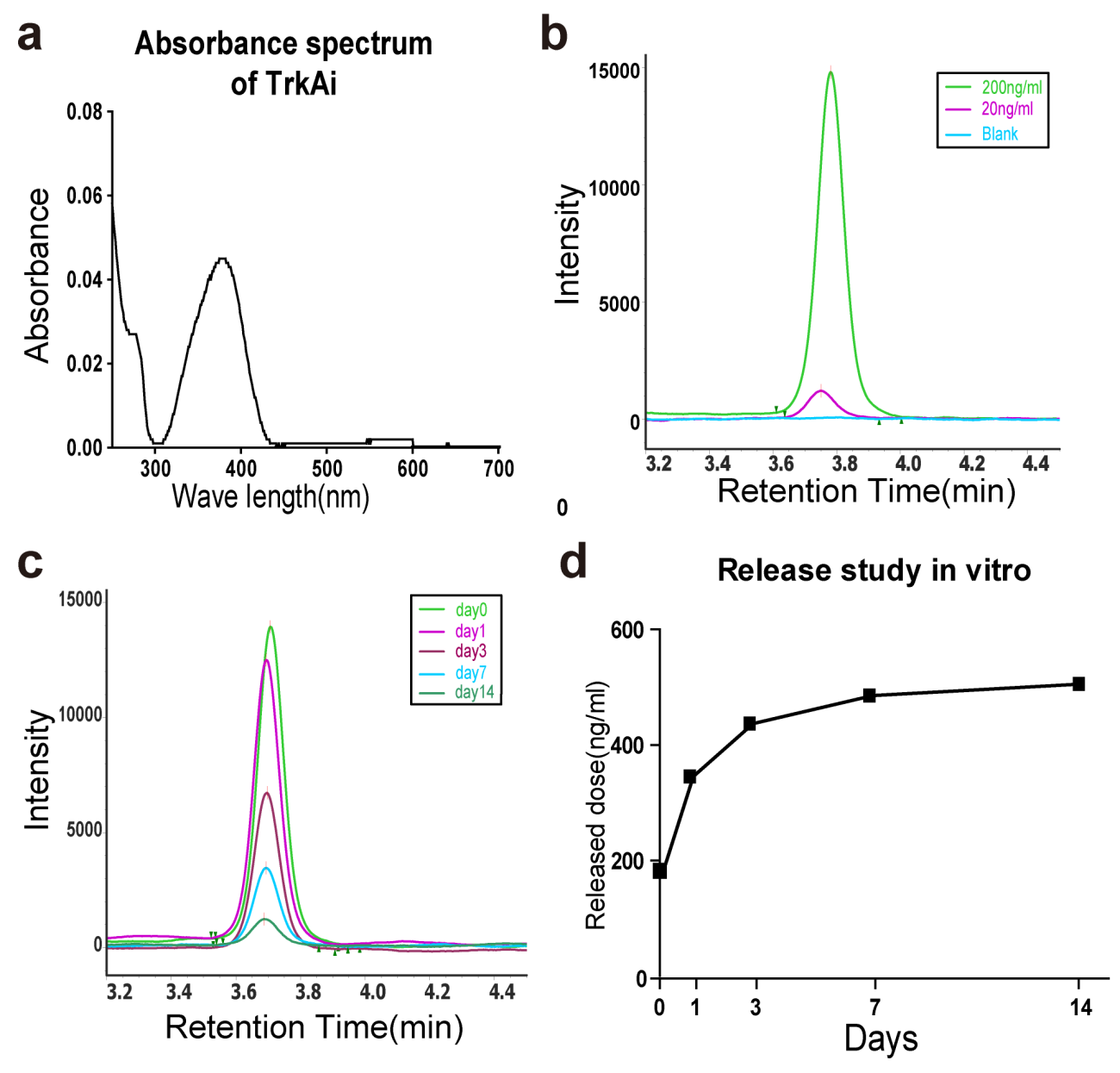
**

**Supplementary Figure 2. HPLC analysis for drug delivery release of PGA-C scaffolds**

(a) The peak of TrkAi absorbance spectrum was 380 nm. (b) The chromatograph peak for TrkAi (200 ng/ml, 20 ng/ml, blank). The calibration curve was calculated with each peak area. (c) The chromatograph peak for TrkAi at various time points (0 d, 1 d, 3 d, 7 d, and 14 d). (d) The release profiles for the PGA-C scaffolds for 14 days. The release of TrkAi slowed down with time but is maintained for over 7 days.


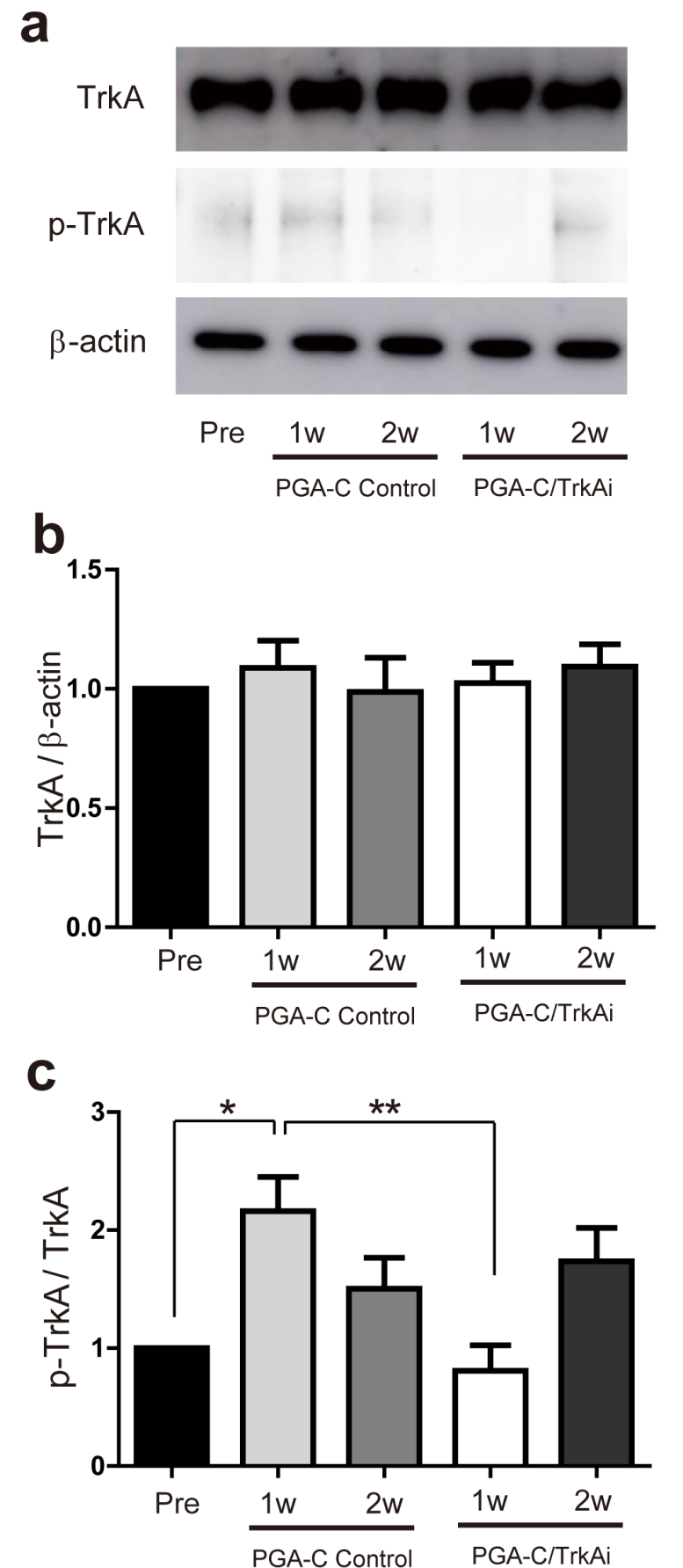


**Supplementary Figure 3. The TrkA inhibitor from PGA-C suppressed phosphorylation of TrkA in vagus ganglion.**

(a) Western blot analysis of TrkA, p-TrkA, and β-actin protein expression in vagus ganglion. Full-length blots/gels are presented in Supplementary Figure 4. (b, c) To evaluate expressions, the intensities of bands were normalized to those of the loading control (β-actin). (b) The expression levels of TrkA were unchanged over time regardless of group. (c) At one week following the treatment with the PGA-C/TrkAi, the phosphorylation level of TrkA was significantly suppressed compared to that of the PGA-C control groups (One-way ANOVA, followed by Bonferroni post hoc test, *p < 0.05, **p < 0.01; n = 4 rats for each. Error bars are SEM).

**
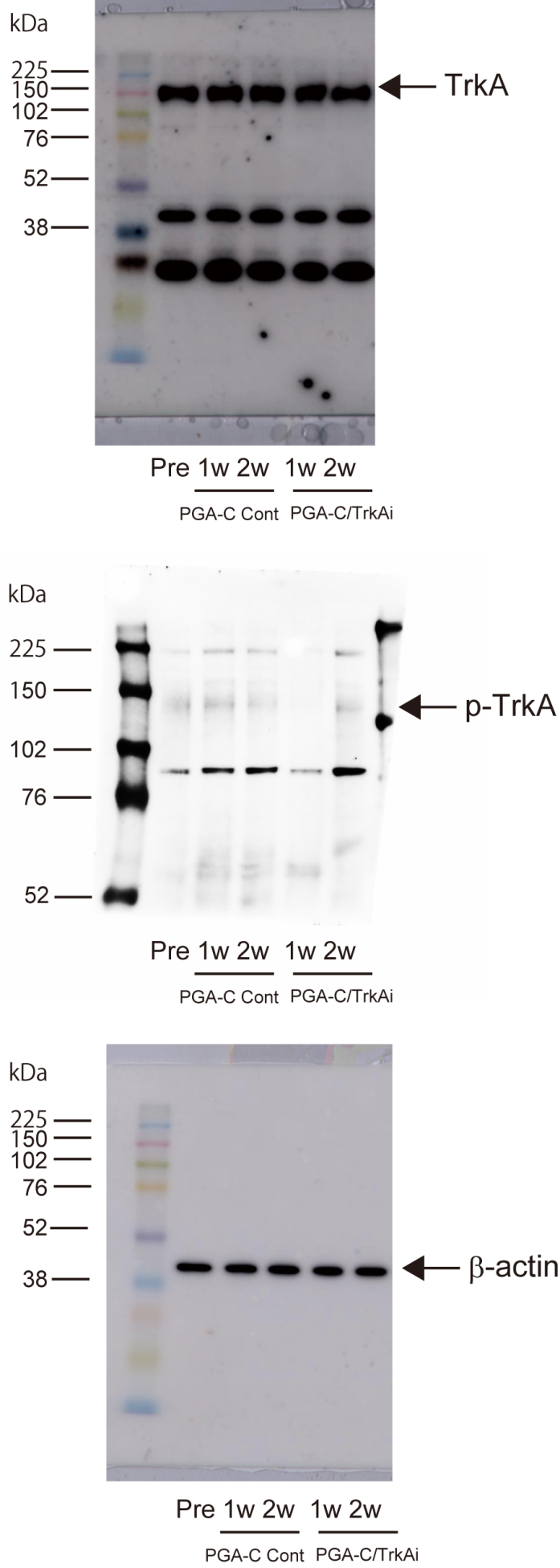
**

**Supplementary Figure 4. Full length blots to accompany Supplemental Figure 3.**

Full length blots corresponding to Supplemental Figures 3a showing TrkA, p-TrkA, and β-actin protein at the point of pre, 1 week and 2 weeks.

**Supplementary Table 1.** The optimized chromatographic conditions for analysis of TrkA inhibitor.

| Drug | Stationary Phase | Mobile Phase | Flow rate (mL/min) | Run time (min) | Injection Volume (µL) | Detection wavelength (nm) | Detection  Limit  (ng/ml) |
| --- | --- | --- | --- | --- | --- | --- | --- |
| TrkA inhibitor | J-Pak Supero C18  (5μm,4.6 × 150 mm) | A:0.1%TFA  B:ACN  A:B=60:40 | 1.000 | 6 | 100 | 380 | 5 |
