# Supplementary material for: TrkA inhibitor promotes motor functional regeneration of recurrent laryngeal nerve by suppression of sensory nerve regeneration
Source: Sci Rep. 2020 Oct 9;10:16892. doi: 10.1038/s41598-020-72288-w (PMC7547101; doi:10.1038/s41598-020-72288-w)
Supplement: Supplementary file 9 — Supplementary Video Legend. [file 41598_2020_72288_MOESM9_ESM.docx]

**Video 1.** Video of laryngeal findings through the laryngoscope in the fixed case (Arytenoid motion angle < 1°). Left arytenoid mobility was not observed.

**Video 2.** Video of laryngeal findings through the laryngoscope in the recovered case (Arytenoid motion angle > 5°). Left arytenoid mobility was observed almost as well as right arytenoid mobility.
